# Assessing the Global Cognition of Community-Dwelling Older Adults Using Motor and Sensory Factors: A Cross-Sectional Feasibility Study

**DOI:** 10.3390/s23177384

**Published:** 2023-08-24

**Authors:** Emilija Kostic, Kiyoung Kwak, Dongwook Kim

**Affiliations:** 1 Department of Healthcare Engineering, The Graduate School, Jeonbuk National University, 567 Baekje-daero, Deokjin-gu, Jeonju 54896, Republic of Korea; emilija.kostic@jbnu.ac.kr; 2 Division of Biomedical Engineering, College of Engineering, Jeonbuk National University, 567 Baekje-daero, Deokjin-gu, Jeonju 54896, Republic of Korea; kykwak@jbnu.ac.kr; 3 Research Center for Healthcare and Welfare Instrument for the Elderly, Jeonbuk National University, 567 Baekje-daero, Deokjin-gu, Jeonju 54896, Republic of Korea

**Keywords:** cognitive function, dementia, PCA, gait, sensory functions

## Abstract

Impairments in gait, postural stability, and sensory functions were proved to be strongly associated with severe cognitive impairment such as in dementia. However, to prevent dementia, it is necessary to detect cognitive deterioration early, which requires a deeper understanding of the connections between the aforementioned functions and global cognition. Therefore, the current study measured gait, postural, auditory, and visual functions and, using principal component analysis, explored their individual and cumulative association with global cognition. The global cognitive function of 82 older Korean males was determined using the Montreal Cognitive Assessment. The motor and sensory functions were summarized into seven independent factors using factor analysis, followed by age and education-level-adjusted linear regression model analysis. The seven factors obtained using factor analysis were gait speed, gait stability, midstance, general auditory ability, auditory recognition, overall visual ability, and postural stability. The linear regression model included years of education, gait stability, postural stability, and auditory recognition, and was able to explain more than half of the variability in cognitive score. This shows that motor and sensory parameters, which are obtainable through wearable sensors and mobile applications, could be utilized in detecting cognitive fluctuations even in the early stages of cognitive deterioration.

## 1. Introduction

Performing even the simplest of daily tasks requires locomotion, balance, hearing, and vision in most cases, and the central nervous system (CNS) is responsible for regulating all of these functions. As we age and as our brain ages, these abilities deteriorate as well [1]. However, in the case of neurodegenerative diseases such as Alzheimer’s dementia (AD), the deterioration in locomotion, balance, hearing, and vision can indicate issues with cognitive function that are not related to the natural aging process, because AD has an effect on the sensory and motor regions of the CNS [2]. Emerging studies have reported a strong relationship of gait, balance, hearing, and vision parameters with global cognitive function. Reduced gait function and gait variability were reported as being related to the incidence of dementia [3,4] and future cognitive decline [5]. In addition, it was shown that among older adults with moderate hearing loss, those with a slow gait had worse global cognitive assessment scores [6]. In the case of postural control, postural instability was found to be correlated with brain atrophy and pathological cognitive impairment in older individuals [7]. Visual and hearing impairments have also been reported to be associated with cognitive decline in older people [8,9,10,11]. By utilizing these motor and sensory function parameters, cognitive deterioration could be detected in the early stages. Such an approach yields more opportunities for slowing or inhibiting the progression of the disease, which would not be available in the case of already progressed dementia.

Most existing studies have used very general methods in obtaining the functional parameters, such as a timed up-and-go test, visual acuity charts, pure tone audiometry, and a one-leg standing test. While these tests are quick and usually used in clinical settings to determine functional impairment, they cannot be considered sensitive enough to observe small quantifiable differences between each participant. So far, many researchers have used functional impairment as a predictor or marker of progressed dementia or a large drop in global cognition. In the case of the beginning stages of cognitive deterioration, however, these binomial parameters are not enough. When it comes to early cognitive impairment, detecting small functional differences may be necessary for the development of diagnostic and prognostic processes. In recent years, the development of wearable sensors and mobile applications has made it easier to obtain various biometric data without the use of expensive or complicated equipment. This allows for more thorough testing even in settings where a quick assessment is needed. For this reason, we opted for more thorough testing methods and obtained a higher number of parameters than what has so far been used to represent gait, postural stability, hearing, and vision in studies related to cognitive deterioration.

Additionally, the aforementioned function parameters are highly correlated with one another within the function they represent, and it may be difficult to determine their independent relationships with the cognitive function. For this reason, an assessment of the correlation between cognition and independent features of gait, postural and sensory functions was performed with the help of linear regression to further investigate the underlying mechanisms that link global cognition to mechanic and sensory functions. By uncovering the correlation between independent mechanics and sensory components and global cognition, we can determine which functional deterioration corresponds to a drop in cognitive ability and hopefully track cognitive deterioration in its early stages. The independent features were extracted using principal component analysis (PCA) because machine learning algorithms converge faster if principal components are used, and dimension reduction provided by PCA prevents overfitting.

To uncover the components that can be used in detecting early cognitive deterioration, the present study examined the functional associations between motor and sensory factors and global cognition, which was achieved by (1) measuring the participants’ cognitive, gait, postural stability, hearing, and visual functions, (2) utilizing PCA to identify independent features, and assessing their correlation to global cognition, and (3) assessing the cumulative association of these features and cognitive function using linear regression.

## 2. Materials and Methods

### 2.1. Demographics and Cognitive Testing

The participants in this study (*n* = 82) were all over the age of sixty-five, male, had no history of dementia diagnosis and could complete daily activities without assistance. They all underwent cognitive, gait, visual, auditory, and postural stability testing. The participants’ heights and weights were measured at the time of testing, and they provided their ages and years of education.

Global cognitive function was examined using the Montreal Cognitive Assessment in Korean (K-MoCA). The K-MoCA is a 30-point test with higher scores representing better cognitive function. The total evaluation time is around 15–20 min, making this test a quick assessment of global cognitive function [12]. Ethical approval to report this study was obtained from the Jeonbuk National University institutional review board (JBNU IRB File No. 2019-09-015-001) and written informed consent was obtained from the participants for their anonymized information to be published in this article. The present study was conducted according to the guidelines of the Declaration of Helsinki.

### 2.2. Gait Assessment

Participants’ gait was recorded using software for capturing human motion (First Principle, Northern Digital Inc., Waterloo, ON, Canada) and motion sensors (Optotrak Certus, Northern Digital Inc., Waterloo, ON, Canada), which captured the position of 17 lower-limb markers. The gait parameters of the participants were obtained as an ensemble average of 3 trials of level walking across a walkway (10 m in length) and were processed using Software for Interactive Musculoskeletal Modeling (SIMM, Motion Analysis Corp., Rohnert Park, CA, USA). Spatiotemporal variables and subdivisions of the gait cycle were used to represent the gait function.

### 2.3. Visual Ability

The Korean standard vision chart, and Lea Numbers chart for contrast sensitivity testing (Lea test intl. LLC, Helsinki, Finland) were used to assess the visual ability of the participants. The best-corrected visual acuity (VA) and corrected contrast sensitivity scores from 1.5 m (CS15M) and 3 m (CS3M) distances were used to represent visual functioning.

### 2.4. Auditory Function

To measure the participants’ auditory function, an audiometer (GSI-61, Grason-Stadler, Copenhagen, Denmark) and the Korean speech audiometry test with pre-recorded samples were used and the participants were seated in a soundproof room, wearing well-adjusted headphones. The variables derived from the data were (1) PTA: an average pure tone audiometry score of the better ear at 0.5, 1, and 2 kHz; (2) SRT: The speech recognition threshold of the better ear; (3) WRS: The word recognition score average; (4) WRSm: Mistake count during word recognition; (5) SRS: The sentence recognition score average; and (6) SRSm: Mistake count during sentence recognition.

### 2.5. Postural Stability

For measuring participants’ postural stability, the Postural Stability Testing mode of the Biodex SD (Biodex Medical System. Inc., Shirley, NY, USA) was utilized. The examination was performed as instructed in the product manual [13]. The obtained variables were three stability indexes, the anteroposterior (APSI), mediolateral (MLSI), and the overall postural stability index (PSI). These indexes represent the amount of swaying of the center of mass while standing and are a reliable measure of postural stability [14].

### 2.6. Statistical Analysis

All of the multiple sensory and gait parameters were standardized and for each function, PCA was performed to derive a small number of uncorrelated independent predictors. Parallel analysis was then performed to determine the significant components of each function. The correlation between the cognitive score and the demographic parameters was assessed using Spearman and Kendall’s tau-b correlation analyses, and the individual components’ correlation with the K-MoCA score was assessed using partial correlation to account for the influence of age and education. Subsequently, a linear regression model containing all of the correlated factors was designed to determine the cumulative association of said factors and the global cognitive function.

Statistical Package for the Social Sciences (SPSS) version 26.0.0 (IBM Corp, New York, NY, USA) was used for all the statistical analyses.

## 3. Results

### 3.1. Demographics and Cognitive Assessment

The cognitive scores and demographic data of the participants (*n* = 82) are presented in Table 1. A significant correlation with the MoCA score was found for age and years of education. Age was found to be negatively correlated (r = −0.226, *p* = 0.005) and education was found to be positively correlated to the MoCA score (r = 0.389, *p* < 0.001). These variables were, therefore, included in the linear regression model when observing the combined motor and sensory function correlations to global cognition.

### 3.2. Gait Assessment

The spatiotemporal variables representing the participants’ gait function are presented in Table 2.

These variables were standardized and PCA was performed, resulting in a total of eleven components. The first three components were able to explain 86.2% of the gait-function variance and parallel analysis revealed that the remaining eight variables did not need to be retained for further analysis. The rotated component matrix, which shows the main variables of each gait-function component (in bold font), is presented in Table 3.

The first component was highly correlated to all the subdivisions of the gait cycle apart from MS and TS, and moderately correlated with cadence, while the third component was highly correlated only to MS and TS. The first component was positively correlated to cadence, SLSP, and SW, and negatively correlated with DLSP, LR, and PS, meaning that the first component has higher values for faster pace, longer single limb support, and shorter double limb support. This component was termed “gait stability” because the duration of the single limb support phase has been referred to as the best index of the limb’s support capability [15]. The second component was highly positively correlated to step length, stride length, and velocity, and was therefore termed “gait speed”.

**Figure 1:**
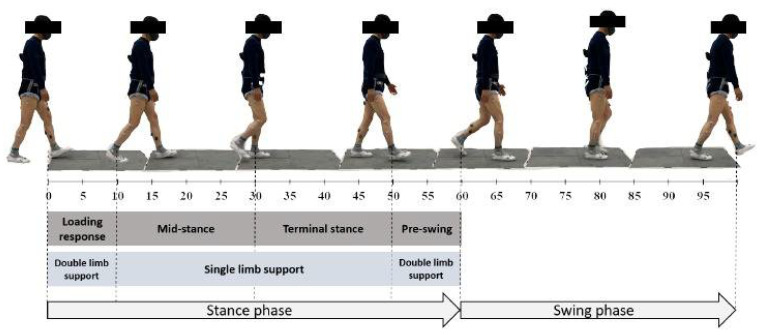
Subdivisions of the gait cycle.

The third component was positively correlated with MS and negatively correlated with TS. In other words, within the single-limb support, the moment at which the midstance phase ends affects the third component (the relations between the subdivisions of the gait cycle can be seen in the illustration presented in Figure 1). The third component was termed “midstance” for this reason. 

Among the three components, gait stability was significantly positively correlated with the K-MoCA score (r = 0.273, *p* = 0.014) after adjusting for age and education.

### 3.3. Visual Ability

The visual ability examination results are presented in Table 4.

After performing PCA, the parallel analysis revealed only one significant component to represent the visual function. This component explained 81.4% of the variance in the data and was highly positively correlated to VA, C3M, and C15M, meaning that the higher value of the visual component represents higher corrected visual ability. The component was found not to be significantly correlated to global cognition. The coefficients of the variables included in the component matrix are presented in Table 4.

### 3.4. Auditory Function

The results of the pure tone audiometry and the speech, word, and sentence recognition tests are shown in Table 5.

PCA and parallel analysis showed that two significant components explained 81.8% of the data variance. Table 5 shows the individual parameters’ effect on the two components.

The first component was highly positively correlated with the WRSm and SRSm and highly negatively correlated with the WRS and SRS scores, meaning that a higher value of the first auditory component is representative of a lower ability for recognizing words and sentences. The second component was highly positively correlated with the PTA and SRT values, meaning that a higher value of the second auditory component is an indicator of lower general auditory ability. After adjusting for age and education, the component representing the inability to recognize words and sentences was negatively correlated with the K-MoCA score (r = −0.375, *p* = 0.001) while the component representing general auditory ability had no significant correlation with cognition.

### 3.5. Postural Stability

The postural stability index scores are presented in Table 6. 

After performing PCA, the parallel analysis revealed only one significant component to represent postural stability. The individual parameters’ effect on the component is presented in Table 6. This component explained 89.1% of the data variance and is highly positively correlated to all the postural indexes, meaning that the higher values of the postural stability component represent more swaying during standing, and consequently lower postural stability. This component representing postural instability was significantly negatively correlated with global cognition (r = −0.394, *p* < 0.001) following age- and education-adjusted analysis.

### 3.6. Global Cognition and Gait, Postural Stability, Auditory and Visual Functions

Among the obtained components, the inability to recognize auditory information, postural instability, and gait stability were significantly linearly correlated to the MoCA score. These components were used in training a linear regression model to determine the cumulative association with the global cognitive function. A model that includes age and education was designed using the stepwise method, where the parameters are added one by one if their coefficients are significant, and removed if they lose significance after the addition of new parameters. The addition criterium was *p* ≤ 0.05 and the removal criterium was p ≥ 0.01. The model parameters are presented in Table 7.

The model excluded age as it was found to be non-significant in explaining the variance in MoCA score with other variables present. Additionally, the MoCA score was observed to be positively associated with the years of education and gait stability while it was negatively associated with the postural instability and inability to recognize auditory information. To summarize, global cognition was shown to be positively associated with years of education, gait stability, postural stability, and auditory recognition. These components together explained 52.7% of the variability in the global cognition scores.

## 4. Discussion

In the present study, the aim was to compare the cognitive function and the independent components that represent gait, postural stability, hearing, and visual ability in a cohort of older adults, and to determine how a combination of these factors is associated with global cognition, to uncover the components that can be used in detecting early cognitive deterioration. Hence, principal component analysis was utilized to extract independent functional components, and their individual and cumulative association with cognitive function was assessed.

The results of the demographics analysis showed that age and years of education were highly correlated with global cognition, which is in accordance with previous reports. Years of education were positively correlated with cognitive scores and education was found to be positively associated with a delay in cognitive decline onset [16]. In addition, the prevalence of dementia was found to be higher in older individuals and those with fewer years of education [17].

Regarding the gait function, the principal component analysis resulted in three significant components: the gait stability component, the gait speed component, and the midstance component. The stability component was found to be significantly correlated with global cognition after adjusting for age and education, while for the gait speed and midstance components, that was not the case. To our knowledge, no previous research has reported a correlation between midstance and cognition. Regarding slow gait, it has been shown to predict future cognitive decline in older individuals [3]; however, it has not been used as a parameter for diagnosing current cognitive impairment. Therefore, it is likely that a decrease in gait speed precedes cognitive deterioration. Whether the correlation between the slowing of the gait and incidence of dementia is causal or a result of a change in a separate, independent factor, is yet to be uncovered. The positive correlation between the stability component and cognitive score indicates that higher stability during walking (represented by a higher percentage of the gait cycle spent relying on one rather than both limbs [15]) is associated with higher global cognition. Similarly, the observed correlation between postural instability and cognitive function was negative, meaning that older individuals with better postural stability perform better on cognitive tests, which is in accordance with previous research [7]. These results show that lower static and dynamic stability correlates to lower cognitive function. It was previously discovered that the incidence of falls was significantly higher among nursing home residents with dementia than among those without dementia [18], suggesting a correlation between stability and severe cognitive impairment. The results of the present research suggest that even in individuals with no incidence of dementia, there is a significant correlation between cognitive ability and static and dynamic stability.

The corrected visual ability component, consisting of visual acuity and contrast sensitivity parameters, was found to be uncorrelated with global cognition. Most of the research examining cognition and visual ability was based on the presence of visual impairment [19,20,21] which was found to be a risk factor for the incidence of dementia. One previous study used cumulative contrast sensitivity scores from five different spatial frequencies [22] and concluded that the risk of cognitive impairment was higher for participants with lower baseline contrast sensitivity scores. Considering the results of previous studies that assessed the visual parameters separately, additional research should be conducted to reach a conclusion regarding the connection between overall visual ability and cognitive function. Examining the contrast sensitivity at more than two spatial frequencies and including the uncorrected vision in the analysis may be necessary to accurately assess the correlation between cognition and visual function.

Higher cognitive scores were correlated with a higher ability to recognize auditory information; however, there was no correlation between cognitive function and general auditory ability. Hearing loss (determined by PTA assessment) has been reported as a risk factor for the incidence of dementia [9,10,23], suggesting that auditory impairment precedes cognitive deterioration, yet it is not correlated to the present cognitive ability. A recent review of the literature concluded that it is most likely that there is a mediating factor between hearing loss and cognition [24]. Regarding auditory recognition, previous research has shown that patients with dementia present with lower sentence comprehension, which makes it difficult to accurately repeat heard sentences [25], suggesting that poor auditory processing may be connected to cognitive impairment. Further research is needed to determine the connections and mechanisms responsible for the observed correlations.

Utilizing the PCA approach made it possible to unite the individual function parameters into independent components that represent specific aspects of each function. Differing from individual measurements, these components are linearly uncorrelated and are a great solution for the problem of multicollinearity, which is common when performing observational medical experiments. Additionally, using PCA to obtain continuous variables allowed more information to be retained than if binomial variables were used. With this method, we were able to determine that while gait speed does not vary with variance in global cognition, stability during gait does. Similarly, although the general auditory ability did not correlate with the cognitive score, the ability to recognize auditory information was significantly correlated with global cognition. In other words, although a method such as PCA is more complicated, it can provide an advantage when distinguishing between normal aging and mild cognitive decline compared to using binomial or individual parameters.

When the individual components were used to design a linear regression model, years of education, gait stability, postural stability, and auditory recognition were found to be significant and were able to explain 52.7% of the cognitive function variability. According to the coefficients in the linear regression model, excluding the years of education which commonly remains constant in late adulthood, the auditory recognition inability component presents the highest association with global cognition, which is followed by postural instability and gait stability. Therefore, a person with high auditory recognition but low overall gait stability may score higher than if the situation were reversed. Similarly, two people with the same auditory recognition ability and gait stability but different postural stability would score differently in terms of cognition. This poses the question of whether a change in one’s auditory recognition, gait stability, or postural stability would correspond to a change in MoCA score. Based on these findings, there is a possibility that the deterioration of any of the aforementioned functions could indicate cognitive deterioration or predict its future occurrence. The motor and sensory assessments at different time points could be used to track general cognition and pose as a warning sign before a significant drop in cognition takes place. The causal relationships are, however, yet to be uncovered.

The main limitation of this study is the small scale. A comparison of gait, postural stability, and sensory functions across the cognitive domains could yield definitive results in the case of a larger-scale study. In addition, this was a cross-sectional study that determined the existence of correlations between the examined functions and cognition but was unable to determine causality, which requires longitudinal research. 

Further research should focus on developing and testing devices or applications for monitoring and determining cognitive status based on a combination of motor and sensory data, which would allow the quick screening and earlier detection of older individuals at a higher risk of developing dementia.

## 5. Conclusions

The current study showed that having more stability during level walking and while standing is positively associated with cognitive function. In addition, auditory recognition ability showed a positive correlation to global cognition. 

Over half of the cognitive function variability in older adults was shown to be explained by the factors presented in this research. This result shows that motor and sensory functional differences correspond to the variability in cognitive function and can be utilized in detecting cognitive fluctuations, even in the early stages of cognitive impairment. 

## Figures and Tables

**Table 1:** Characteristics of the participants.

Variable	MEAN	SD	MIN	MAX
Age (years)	73.9	4.4	65.0	85.0
Height (cm)	167.2	5.7	153.0	180.0
Weight (kg)	67.0	8.3	46.5	88.8
Years of education	13.6	3.3	6.0	23.0
K-MoCA score	22.0	3.8	12.0	29.0

SD, Standard Deviation.

**Table 2:** Gait function parameters of the participants.

Variable	MEAN	SD	MIN	MAX
Step length (cm)	65.0	5.8	51.8	82.7
Stride length (cm)	125.9	9.8	99.5	149.8
Cadence (steps/min)	110.0	7.7	86.9	128.2
Gait velocity (cm/s)	115.7	14.5	77.5	153.0
SLSP (%)	39.3	1.3	35.5	42.2
DLSP (%)	22.1	2.2	17.7	28.6
LR (%)	10.8	1.4	7.7	15.8
MS (%)	20.7	2.4	12.7	26.5
TS (%)	18.6	2.6	11.7	27.8
PS (%)	11.2	1.1	9.0	13.4
SW (%)	38.6	1.3	35.8	41.2

SD, Standard Deviation; SLSP, Single-Limb-Support Portion; DLSP, Double-Limb-Support Portion; LR, Loading Response; MS, Mid-Stance; TS, Terminal Stance; PS, Pre-Swing; SW, Swing.

**Table 3:** Component matrix of the gait function.

Variable	Component		
	PC1	PC2	PC3
DLSP (%)	**−0.990**	−0.100	−0.008
LR (%)	**−0.916**	−0.064	−0.153
SLSP (%)	**0.872**	−0.105	−0.159
PS (%)	**−0.862**	−0.120	−0.179
SW (%)	**0.797**	0.274	0.170
Cadence (steps/min)	**0.660**	0.379	0.102
Step length (cm)	0.339	**0.890**	−0.027
Stride length (cm)	0.372	**0.884**	−0.019
Gait velocity (cm/s)	0.601	**0.758**	0.038
TS (%)	0.181	−0.027	**−0.979**
MS (%)	0.282	−0.030	**0.934**

DLSP, Double-Limb-Support Portion; LR, Loading Response; SLSP, Single-Limb-Support Portion; PS, Pre-Swing; SW, Swing; TS, Terminal Stance; MS, Mid-Stance; PC, Principal Component.

**Table 4:** Visual ability of the participants.

Variable	MEAN	SD	MIN	MAX	PC1
CS3M	16.1	5.6	0.0	25.0	0.892
CS15M	22.2	4.1	5.0	25.0	0.944
VA	0.8	0.3	0.2	2.0	0.870

SD, Standard Deviation; CS3M, Contrast Sensitivity at 3 m distance; CS15M, Contrast Sensitivity at 1.5 m distance; VA, Visual Acuity; PC, Principal Component.

**Table 5:** The auditory function of the participants.

Variable	MEAN	SD	MIN	MAX	PC1	PC2
PTA (dB HL)	26.3	11.8	9.2	61.7	0.305	**0.901**
SRT (dB HL)	22.6	11.8	5.0	57.5	0.088	**0.884**
WRS	75.7	13.2	32.0	96.0	**−0.731**	−0.463
WRSm	12.2	6.5	2.0	34.0	**0.744**	0.449
SRS	97.3	3.3	81.2	100.0	**−0.930**	0.018
SRSm	2.1	2.7	0.0	15.0	**0.920**	−0.039

SD, Standard Deviation; PTA, Pure Tone Audiometry better ear 0.5, 1, and 2 kHz average; SRT, Speech Recognition Threshold; WRS, Word Recognition Score; WRSm, Word Recognition mistakes; SRS, Sentence Recognition Score; SRSm, Sentence Recognition mistakes; PC, Principal Component.

**Table 6:** Postural stability of the participants.

Variable	MEAN	SD	MIN	MAX	PC1
PSI	0.8	0.5	0.3	3.4	**0.995**
APSI	0.6	0.4	0.2	2.5	**0.937**
MLSI	0.3	0.3	0.1	2.1	**0.897**

SD, Standard Deviation; PSI, Postural Stability Index; APSI, AnteroPosterior Stability Index; MLSI, MedioLateral Stability Index; PC, Principal Component.

**Table 7:** Age- and education-adjusted linear model significant parameters.

Component	β	S.E.	t	*p*-Value	R^2^
Auditory Recognition Inability	−0.249	0.349	−2.684	0.009	0.527
Postural instability	−0.282	0.334	−3.179	0.002	
Gait stability	0.191	0.301	2.386	0.019	
Years of education	0.411	0.093	5.058	<0.001	

S.E., Standard Error.

## Data Availability

The data presented in this study are available as Supplementary Material.

## Supplementary Materials

The following supporting information can be downloaded at: https://www.mdpi.com/article/10.3390/s23177384/s1, Dataset S1: manuscript-dataset.csv.
